# Prevalence of Cardiovascular Risk Factors among Young Adults (18–25 Years) in Mozambique

**DOI:** 10.3390/jcdd10070298

**Published:** 2023-07-12

**Authors:** Isa Silva, Albertino Damasceno, Filipa Fontes, Natália Araújo, António Prista, Neusa Jessen, Patrícia Padrão, Carla Silva-Matos, Nuno Lunet

**Affiliations:** 1 EPIUnit—Instituto de Saúde Pública, Universidade do Porto, 4050-600 Porto, Portugal; 2 Laboratório para a Investigação Integrativa e Translacional em Saúde Populacional (ITR), Universidade do Porto, 4050-600 Porto, Portugal; 3 Departamento de Medicina, Faculdade de Medicina, Universidade Eduardo Mondlane, Maputo 1100, Mozambique; 4 Departamento de Ciências da Saúde Pública e Forenses e Educação Médica, Faculdade de Medicina da Universidade do Porto, 4200-450 Porto, Portugal; 5 Unidade de Investigação em Enfermagem Oncológica, Centro de Investigação do Instituto Português de Oncologia do Porto, 4200-072 Porto, Portugal; 6 Faculdade de Ciências de Educação Física e Desporto, Universidade Pedagógica de Maputo, Maputo 1100, Mozambique; 7 Faculdade de Ciências da Nutrição e Alimentação, Universidade do Porto, 4099-002 Porto, Portugal; 8 Unidade de Gestão do Fundo Global—Direção de Planificação e Cooperação, Ministério da Saúde de Moçambique, Maputo 1100, Mozambique

**Keywords:** Africa, Mozambique, cardiovascular diseases, risk factors, prevalence

## Abstract

The life course development of cardiovascular diseases (CVDs) and the undergoing epidemiological transition in Mozambique highlight the importance of monitoring the cardiovascular risk profile in young adults. Therefore, this study aims to estimate the prevalence of CVD risk factors in a population aged 18–25 years living in Mozambique. A total of 776 young adults from a nationally representative sample were evaluated in 2014/2015 following the World Health Organization’s STEPwise approach to chronic disease risk factor surveillance. Current smoking was the most prevalent among rural men (10.8%, 95%CI: 6.3–17.8), and drinking was most prevalent among urban men (38.6%, 95%CI: 29.3–48.8). The proportion of young adults not engaging in at least 75 min of vigorous physical activity per week ranged between 14.5% in rural men and 61.6% in urban women. The prevalence of being overweight/obese and hypertension were highest among urban women (21.6%, 95%CI: 14.7–30.6) and urban men (25.2%, 95%CI: 15.9–37.6), respectively. Education >8 years (vs. none) was independently associated with lower odds of being a current smoker, and increased monthly household income was associated with increased odds of low levels of physical activity. This study shows that important CVD risk factors are already common in the young adult population of Mozambique.

## 1. Introduction

Cardiovascular diseases (CVDs) are the leading cause of death worldwide. In 2020, approximately 19.1 million deaths were attributed to CVD globally, with sub-Saharan Africa (SSA) being one of the regions with the highest mortality rates [1]. CVDs, like other noncommunicable diseases (NCDs), are related to behavioral factors such as tobacco and alcohol use, physical inactivity, and other factors that interact in complex ways with these behavioral factors, such as obesity and hypertension [2,3]. According to the 2019 Global Burden of Disease data [4], dietary risks, high systolic blood pressure, elevated body mass index (BMI), low levels of physical activity, and tobacco and alcohol use are the leading causes of disability-adjusted life years (DALYs) in developing countries. Moreover, over the past decade, their frequency has been increasing in this context [4]. 

CVDs were shown to have a life course evolution, with exposure to its main risk factors starting during young adulthood or even earlier [2,5,6], and there is a robust association between maintaining a healthy lifestyle during young adulthood and the presence of a low CVD risk profile in adult life [7,8,9,10]. Therefore, targeting young adults for primary prevention of CVD risk factors is essential to reduce the risk of CVD later in life [11,12]. 

Mozambique is undergoing demographic and epidemiologic transitions [13,14,15], and, like in other SSA countries, competing demands to address infectious diseases and poverty pose challenges to CVD prevention and risk factor management. In addition, CVD in SSA countries seems to disproportionally affect the younger and more productive population compared to the rest of the world [16]. This highlights the importance of monitoring the current cardiovascular risk profile of young adults. Therefore, the present study aims to describe the prevalence of CVD risk factors, namely tobacco use, alcohol consumption, hypertension, being overweight/obese, and physical inactivity, among young adults in Mozambique.

## 2. Materials and Methods

### 2.1. Study Design

This study is based on a cross-sectional evaluation conducted between December 2014 and February 2015 in a nationally representative sample of the Mozambican population. The study protocol was approved by the National Bioethics Committee for Health (98/CNBS/14).

### 2.2. Selection of Participants

The study implementation and sampling strategy have been described in detail elsewhere [15,17]. Participants were selected from a multistage sampling procedure based on 2007 census data [18] to be representative at the national and provincial levels, according to residence in urban or rural areas. Homeless people and people living in collective residential institutions (e.g., hotels, hospitals, and military facilities) were not eligible to participate. Sampling procedures began with the selection of 120 primary sampling units (geographical units including 400 to 600 households) with probability proportional to the number of households and units stratified by province, urban/rural areas, and socioeconomic strata. Within each primary sampling unit, one enumeration area (geographical unit with 80 to 150 households) was then randomly selected. Within each primary sampling unit, 24 households were randomly and systematically selected from updated household lists. All dwellers aged 15 to 64 years from each household were listed, and a maximum of two individuals were selected whenever available (one aged 15 to 44 years and one aged 45 to 64 years); if there was more than one household member in each of these age groups, only one individual per group was randomly selected using a Kish selection grid. A total of 3277 individuals were invited, of whom 3119 agreed to participate in the survey. Sampling weights were computed considering the number of participants evaluated in each stratum relative to the expected number per stratum according to the population projection for the same period. The present analysis includes 776 participants aged 18–25 years.

### 2.3. Evaluation of the Participants 

Subjects were evaluated using standardized methods according to the World Health Organization’s (WHO) STEPwise approach to chronic disease risk factor surveillance (STEPS). The Portuguese version of the WHO STEPS instrument for NCD risk factors (Core and Expanded Version 3.0) [19] was used for data collection by trained interviewers. 

Demographic information and behavioral measures, namely tobacco use, alcohol consumption, and physical activity, were collected during the interview, along with measurements of physical features (body weight, waist circumference, and blood pressure). The classification of residence as urban or rural and the definition of the highest level of education attained were in accordance with 2007 census data [18].

Participants were asked if they currently smoked any type of tobacco product, such as cigarettes, cigars, or pipes, and were considered current smokers if they answered in the affirmative. Those who were smokers were asked about the type of tobacco smoked (manufactured and/or hand-rolled).

To assess alcohol consumption, participants were asked if they had consumed any alcohol in the past 30 days, and if so they were classified as current drinkers; if not, they were classified as not current drinkers. For current drinkers, exposure to binge drinking was considered whenever they reported having consumed six or more standard drinks on a single drinking occasion in the past 30 days [19]. In addition, current drinkers were asked whether they had consumed traditional beverages (e.g., homebrewed alcohol) in the past seven days. 

The Global Physical Activity Questionnaire (GPAQ), as part of the STEPS instrument, was used to assess physical activity. Participants were asked if they engaged in vigorous and moderate work and recreation activities and the number of days they engaged in each activity in a typical week, as well as the amount of time they spent on each activity on a typical day when applicable. Travel-related activities included moderate-intensity activities, such as walking and cycling continuously for at least 10 min. To describe the physical activity of this population, the categorical indicator of the recommended amount of physical activity for health according to the WHO and the cut-off point of at least 75 min of vigorous physical activity were used [19]. Those respondents doing, per week, less than 150 min of moderate-intensity physical activity, less than 75 min of vigorous-intensity activity, or an equivalent combination of moderate- and vigorous-intensity physical activity achieving less than 600 MET (metabolic equivalent) minutes were considered as not meeting the WHO recommendations [20]. For the calculation of an equivalent combination of moderate- and vigorous-intensity physical activity, respective MET values of 4 and 8 were used [19]. 

Body weight was measured to the nearest 0.1 kg with a digital scale, and height was measured to the nearest 0.1 cm while standing with a portable stadiometer, with participants wearing light clothing and no footwear. BMI was calculated as weight (in kilograms) divided by the square of height (in meters), and participants were considered as being overweight/obese when BMI ≥ 25.0kg/m^2^, as defined by the WHO [21]. Waist circumference was measured to the nearest 0.1 cm using a tape measure with constant tension that was placed directly on the skin or over light clothing at the level of the midpoint between the lower edge of the last rib and the iliac crest in the mid-axillary line. Participants were classified as having abdominal obesity if their waist circumference was greater than 88 cm for women and 102 cm for men [21]. 

Blood pressure was measured by nonphysician-trained interviewers using a semiautomatic sphygmomanometer (Bosch & Sohn Medicus UNO, Germany) with a universal cuff of 22–42 cm. After a 5 min rest period, blood pressure was measured on the right arm three times at 3 min intervals while the patient was sitting comfortably [19]. The mean of the first two measurements was used for analysis if they differed by 10 mmHg (millimeters of mercury) or less for both systolic (SBP) and diastolic blood pressure (DBP), otherwise the mean of the last two measurements was used. Arterial hypertension was defined as systolic blood pressure (SBP) ≥ 140 mmHg, diastolic blood pressure (DBP) ≥ 90 mmHg, or antihypertensive drug therapy in the last two weeks, as well as the combination of any of these indicators [22]. Hypertensive patients were considered aware of hypertension whenever having been told by a health professional, in the previous 12 months, that they had hypertension or high blood pressure, or whenever reporting a pharmacological treatment for hypertension. Participants reporting having used antihypertensive medication in the previous two weeks were considered as being treated pharmacologically for hypertension. Control of hypertension was defined as SBP less than 140 mmHg and DBP less than 90 mmHg among treated hypertensive patients [17].

### 2.4. Statistical Analysis 

Sex- and place of residence-specific prevalence estimates with 95% confidence intervals (95%CI) were computed for all risk factors. Estimates by type of tobacco consumed were computed for current smokers. Drinking of traditional beverages and binge drinking were estimated among current drinkers. Awareness, treatment, and control of hypertension were estimated among hypertensives. Sex-, education- and income-adjusted odds ratios (ORs) and corresponding 95%CIs were computed using logistic regression to estimate the strength of the independent association between sociodemographic characteristics and risk factor variables. All analyses were conducted with STATA version 15.1 considering the sampling weights and adjusted for strata and clustering at the primary sampling unit level.

## 3. Results

Just over 55% of this population lived in rural areas. In total, 18.6% of women and 7.9% of men had no formal education, and 2.6% of women and 4.2% of men had more than 12 years of education. About one-third of the sample reported having a monthly household income of less than USD 15 per adult household member aged 18 years or older (Table 1).

### 3.1. Prevalence of Risk Factors According to Sex and Place of Residence

**Figure 1:**
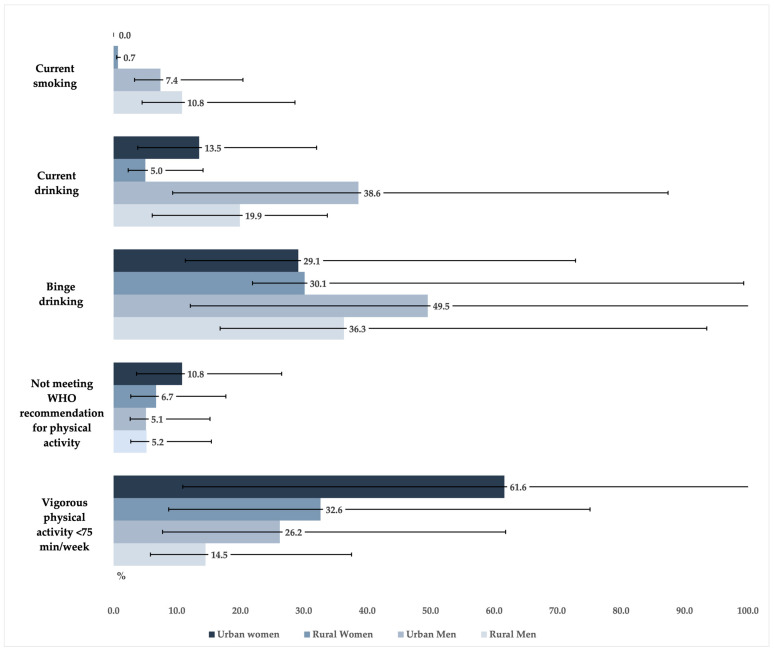
Prevalence of current smoking, current drinking, binge drinking (among current drinkers), not meeting World Health Organization (WHO) recommendation for physical activity [20], and vigorous physical activity <75 min/week in a sample of young adults aged 18–25 years from Mozambique by sex and place of residence and the corresponding 95% confidence intervals.

Figure 1 shows that current smoking was most prevalent among rural men (10.8%) and that drinking was most prevalent among urban men (38.6%). Manufactured cigarettes were the most common type of smoked tobacco, with hand-rolled cigarettes being consumed by 33.5% (95%CI: 11.9–65.4) of rural men and 5.3% (95%CI: 0.7–31.5) of urban men. Among current drinkers, nearly half of urban men and about one-third of rural men, urban women, and rural women reported binge drinking. The percentage of the population that did not meet the WHO recommendations for physical activity was lower than 11%, regardless of sex and place of residence. However, the proportion of young adults not engaging in at least 75 min of vigorous physical activity per week ranged between 14.5% among rural men and 61.6% among urban women.

**Figure 2:**
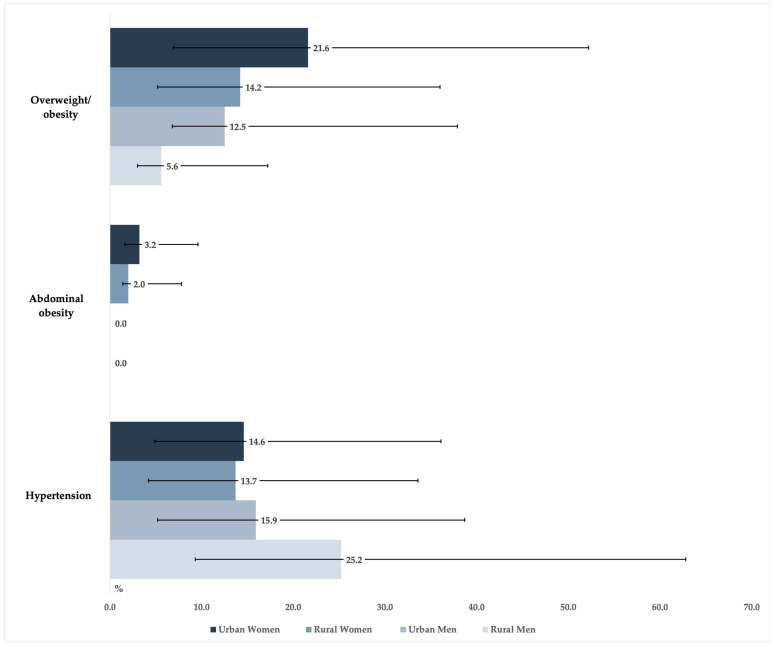
Prevalence of being overweight/obese, abdominal obesity, and hypertension in a sample of young adults aged 18–25 years from Mozambique by sex and place of residence and the corresponding 95% confidence intervals.

The prevalence of being overweight/obese was higher in women, ranging from 5.6% among rural men to 21.6% among urban women. Abdominal obesity was observed only among women (urban: 3.2%; rural: 2.0%). Hypertension was more common in men from rural areas (25.2%) and ranged between 13.7% and 15.9% in the remaining groups (Figure 2). Among hypertensives, women and men from urban areas were the most aware of their condition (15.7% (95%CI: 6.4–33.4) and 6.3% (95%CI: 1.2–20.1), respectively), and only 1.9% of rural women and none of the rural men were aware of this condition. Hypertension treatment and control were observed only among urban women (8.4% (95%CI: 2.8–22.2) and 5.4% (95%CI: 1.5–18.0), respectively).

### 3.2. Association between the Sociodemographic Characteristics and the Risk Factors

Table 2 and Table 3 present the independent association between sociodemographic characteristics and each CVD risk factor. Men presented significantly higher odds of smoking (OR = 32.42, 95%CI: 8.21–128.04), alcohol drinking (OR = 3.94, 95%CI: 2.61–5.95), and hypertension (OR = 2.09, 95%CI: 1.20–3.63), while the opposite was observed for not engaging in at least 75 min of vigorous physical activity per week (OR = 0.28, 95%CI:0.18–0.44) and for being overweight/obese (OR = 0.43, 95%CI: 0.21–0.87), which were more likely among women. Urban dwellers were more likely to be current alcohol drinkers (OR = 2.36, 95%CI: 1.36–4.10) and not to engage in at least 75 min of vigorous physical activity per week (OR = 1.91, 95%CI: 1.06–3.45). Smoking and hypertension were gradually less likely to be observed as the level of education increased, while not meeting the WHO recommendations regarding physical activity was increasingly more frequent with increasing income. The proportion of current drinkers reporting the consumption of traditional beverages was highest in rural areas (men: 50.3%, 95%CI: 26.8–73.7; women: 13.8%, 95%CI: 3.2–43.2), though also observed in the urban setting (men: 13.2%, 95%CI: 6.4–25.0; women: 5.3%, 95%CI: 1.5–16.9). 

## 4. Discussion

This study, conducted in a population of young adults from Mozambique, describes the prevalence of important CVD risk factors considering sociodemographic characteristics. In general, young men had a higher prevalence of alcohol consumption and binge drinking, smoking, and hypertension, while women were more physically inactive and overweight/obese. Being overweight/obese, current drinking, and low levels of physical activity prevailed in the urban setting. 

Tobacco use usually starts during young adulthood or earlier, possibly due to the influence of parents/grandparents or peers [23,24] and, more recently, due to the influence of social media, which specifically entices young people [25,26]. As reported in previous studies from other sub-Saharan countries [23,24,25], tobacco use was more prevalent among young adults from rural areas and among less educated dwellers. In addition, education >8 years (vs. none) was independently associated with lower odds of being a current smoker, which might be explained by the fact that more educated individuals seem to be more aware of the harmful effects of tobacco use [27,28]. Increasing taxation on tobacco prices has made this practice more prevalent among those with higher economic power [29], although our study was not conclusive in this feature. In Mozambique, tobacco control policy is regulated by Government Decree 11/2007. It is prohibited to smoke in public places, to sell tobacco products in educational and health institutions, and to sponsor, support, or collaborate with the tobacco industry in public health campaigns. There is also a ban on tobacco advertising on social media, billboards, and public transportation terminals. However, all these measures still do not meet the requirements of the WHO Framework Convention on Tobacco Control and its guidelines [30,31]. To reduce tobacco use among young people, effective laws must continue to be enforced that comprehensively ban all forms of direct tobacco advertising.

The Global Burden of Disease 2020 [32] concludes that the majority of the world’s population that are consuming harmful amounts of alcohol are young adults, especially young men, which is consistent with our findings that men are more likely to be current alcohol users. Our study revealed a higher prevalence of current drinking and binge drinking among young adults from urban areas, while more frequent consumption of traditional beverages was found among rural young adults, which can be explained by the fact that young adults in urban areas mainly drink alcohol produced by the official alcohol industry, whereas in rural areas they mainly drink homebrewed alcohol, as it is cheaper and more readily available than officially produced alcohol [33]. Studies from South Africa and Kenya found that binge drinking was more frequent in young adults (19.6% and 35.2% among drinkers, respectively) [34,35]. Moreover, binge drinking is widespread in the WHO African Region, particularly among persons aged 20 to 24 years (50.7% of drinkers), and carries a high potential for health harms, including increased risk of CVD and social harm [36]. Our findings highlight the importance of developing efforts to prevent alcohol consumption at this stage of life, especially among young men. 

Not complying with recommendations from the WHO for physical activity was relatively infrequent in this population, with the highest proportions identified in women and urban areas. Following this result, a study from urban South Africa also verified that young women tend not to engage in regular physical activity [37]. The proportion of women, especially in urban areas, who do not engage in at least 75 min of vigorous physical activity per week was also high. This could be explained by a decrease in the involvement of young adults in agricultural activities, especially in urban areas, as this is one of the activities in which women’s contribution was traditionally strong in Mozambique [38]. Additionally, our study found an association between a higher household income and noncompliance to WHO recommendations. Although we analyzed household instead of individual income, this may reflect more sedentary occupations and that less physical effort is needed to ensure basic household needs among those with higher income, such as the expenditure to fetch water or lower travel-related physical activity. Nevertheless, according to Lear et al. [39], recreational and non-recreational physical activity was associated with a lower risk of mortality and CVD events in individuals from low to high-income countries. Therefore, measures should be taken to help young adults engage in regular and effective physical activity to reduce CVD risk.

The proportion of participants identified as overweight/obese in this study is consistent with previous data from younger age groups in Mozambique [40]. Our study also found a higher prevalence in women, regardless of place of residence, which is again consistent with previous data. Although biological differences in the prevalence of obesity in men and women have been described in the literature, social and cultural gender differences that might contribute to being overweight/obese have also been pointed out [41,42]. Differences were found between urban and rural areas, with urban areas having a higher prevalence of being overweight/obese. Urbanization is associated with the adoption of unhealthy lifestyle habits, such as increased consumption of highly processed foods and sugar-sweetened beverages and lower levels of physical activity [43], which is also consistent with our findings of lower levels of vigorous-intensity physical activity in the urban setting. Our results showed a relationship between higher household income and overweight/obesity that did not reach significance after adjustment. Nevertheless, an increased tendency in overweight/obesity with increasing household income was observed in this population, as in previous studies in Mozambique using STEPwise approach in the population aged 25-64 years [14,44]. No abdominal obesity was found in young men, and a markedly low percentage was found in women from both settings. However, cut-off points to define a subject as obese can be influenced by ethnicity, among other factors [45], and may not be the most appropriate for this population. 

The high prevalence of hypertension in young adults in Mozambique compels a reflection on the main determinants of high blood pressure in this population. Of these, excessive salt intake stands out and needs to be prevented and controlled from a young age. This is likely to be a reflection of the rising consumption of highly processed foods [43], along with the changing dietary trends in Mozambique [14,44]. In our study, the likelihood of developing hypertension was higher among the less educated. This is consistent with the findings of representative household surveys from 76 low- and middle-income countries, which concluded that the least educated had a higher prevalence of hypertension [46]. In accordance with two systematic reviews including data from sub-Saharan countries and young adults [47], the prevalence of hypertension in our study was higher in rural dwellers, and the awareness of hypertension was higher among young adults in urban areas [44]. Furthermore, low levels of treatment and less control of hypertension are described in the general population, with most studies revealing better prevalence among women [48], which is reflected in our results where only young women in urban areas were treated for and controlled their hypertension. One possible explanation for this result is the increased chance of women having their blood pressure measured during pregnancy.

The present study used a standardized method to monitor NCD risk factors that will allow future comparisons. To our knowledge, this is the first study to provide a detailed characterization of the cardiovascular risk profile based on a representative sample of the young adult population of Mozambique. Nevertheless, studies with larger samples and more precise prevalence estimates for this population are needed, as well as a systematic assessment of lifestyles and physical measures.

## 5. Conclusions

Our findings show that important risk factors for CVD are prevalent in the young adult population (18–25 years) in Mozambique. This highlights the need for continuous monitoring of these risk factors and the development of culturally appropriate interventions for this population. It is important to strengthen tobacco control measures in line with the WHO Framework Convention on Tobacco Control, as well as to promote healthy eating, reductions in alcohol consumption, and adequate levels of physical activity in young adults in Mozambique. In this context, the number of nutrition professionals has been increasing, physical activity is being promoted in schools, selling alcohol to minors is prohibited, and mass media outlets are highlighting the importance of healthy lifestyles. Additionally, the Ministry of Health has issued guidelines for the diagnosis and treatment of hypertension and diabetes. 

## Figures and Tables

**Table 1:** Sociodemographic characteristics of the participants (*n* = 776).

	Women (*n* = 488)			Men (*n* = 288)		
	*n*	Unweighted (%) ^1^	Weighted (%) ^2,3^	*n*	Unweighted (%) ^1^	Weighted (%) ^2,3^
Place of residence						
Urban	250	51.2	44.8	162	56.3	44.2
Rural	238	48.8	55.2	126	43.8	55.8
Education (years)						
None	80	16.4	18.6	22	7.6	7.9
1–4	121	24.8	28.9	47	16.3	18.6
5–7	68	13.9	14.8	69	24.0	26.6
8–10	129	26.4	23.5	73	25.4	25.0
11–12	72	14.8	11.6	61	21.2	17.7
>12	18	3.70	2.60	16	5.60	4.20
Monthly income (USD/household member aged ≥18 years)						
0–15	116	29.5	32.2	76	20.5	35.2
16–22	81	30.6	227	36	14.5	17.6
23–44	83	21.1	19.3	48	19.3	17.8
45–89	60	15.3	13.8	39	15.7	13.3
≥90	53	13.5	12.0	50	20.1	16.0

^1^ The sum of the number of participants in the income category is lower than 488 for women and 288 for men due to missing data. ^2^ The sum of the proportions within each category may not be 100% because of rounding. ^3^ The sampling weights were computed considering the number of participants evaluated in each stratum in relation to the number of participants expected per stratum according to the population projections for the same period.

**Table 2:** Prevalence and corresponding 95% confidence interval of current smoking, current drinking, binge drinking (among current drinkers), not meeting World Health Organization (WHO) recommendation for physical activity [20], and vigorous physical activity <75 min/week in a sample of young adults aged 18–25 years from Mozambique; ORs and 95% confidence intervals of the association between sociodemographic characteristics and the previous variables are included.

	Current Smoking		Current Drinking		Binge Drinking ^2^		Not Meeting WHO Recommendation Physical Activity ^3^		Vigorous Physical Activity <75 min/Week ^4^	
	% (95%CI)	OR ^1^ (95%CI)	% (95%CI)	OR ^1^ (95%CI)	% (95%CI)	OR ^1^ (95%CI)	% (95%CI)	OR ^1^ (95%CI)	% (95%CI)	OR ^1^ (95%CI)
Sex ^5^										
Women	0.3 (0.1–1.3)	ref	8.8 (6.7–11.5)	ref	29.6 (17.6–45.4)	ref	8.5 (6.2–11.6)	ref	45.5 (37.8–53.4)	ref
Men	9.3 (6.2–13.7)	32.42 (8.21–128.04)	28.2 (22.6–34.5)	3.94 (2.61–5.95)	44.4 (34.0–55.3)	1.59 (0.61–3.71)	5.1 (3.1–8.3)	0.64 (0.34–1.20)	19.6 (14.7–25.8)	0.28 (0.18–0.44)
Place of residence ^5^										
Rural	4.6 (2.8–7.3)	ref	10.7 (7.5–15.2)	ref	34.7 (20.6–52.1)	ref	6.1 (4.0–9.2)	ref	25.7 (19.6–32.9)	ref
Urban	2.8 (1.5–5.2)	1.00 (0.34–2.90)	23.0 (17.2–30.0)	2.36 (1.36–4.10)	41.9 (32.5–51.8)	1.59 (0.66–3.84)	8.6 (6.1–12.1)	0.84 (0.40–1.77)	48.2 (39.7–56.9)	1.91 (1.06–3.45)
Education (years) ^5^										
None	7.4 (3.8–14.3)	ref	6.9 (3.9–12.2)	ref	28.7 (8.4–63.8)	ref	4.3 (1.4–12.7)	ref	36.1 (23.8–50.5)	ref
1–7	4.4 (2.4–8.2)	0.26 (0.08–0.87)	13.2 (8.9–19.0)	1.32 (0.58–2.98)	43.3 (28.4–59.6)	1.69 (0.31–9.32)	6.3 (4.0–9.6)	2.46 (0.71–8.56)	28.8 (21.9–36.9)	0.71 (0.35–1.44)
>8	1.7 (0.8–3.7)	0.06 (0.02–0.25)	22.7 (18.0–28.3)	1.67 (0.75–3.72)	37.9 (26.6–50.6)	1.20 (0.21–6.74)	9.3 (6.6–13.1)	3.03 (0.76–12.14)	42.9 (36.0–50.2)	1.02 (0.48–2.16)
Monthly income (USD/household member aged ≥18 years) ^5^										
0–15	5.3 (2.7–10.0)	ref	17.3 (12.4–23.5)	ref	44.3 (26.2–64.0)	ref	4.5 (4.5–8.1)	ref	34.0 (25.8–43.6)	ref
16–44	4.3 (2.2–8.1)	0.85 (0.28–2.57)	16.1 (11.5–22.1)	0.91 (0.55–1.49)	33.8 (22.0–48.1)	0.67 (0.27–1.60)	6.8 (4.5–10.4)	1.52 (0.67–3.46)	33.8 (27.3–40.9)	0.85 (0.55–1.33)
≥45	3.1 (1.4–6.7)	0.88 (0.28–2.83)	21.9 (15.7–29.6)	1.02 (0.58–1.79)	43.7 (28.4–60.3)	0.96 (0.33–2.84)	11.4 (7.5–17.0)	2.53 (1.08–5.91)	44.5 (36.4–53.0)	1.36 (0.83–2.23)

ref: Reference category. ^1^ Derived from models including sex, place of residence, education, and income. ^2^ Defined if participant reported the consumption of six or more standard drinks on a single drinking occasion in the past 30 days [19]. ^3^ Defined according to the WHO 2020 recommendations on physical activity and sedentary behavior [20]. ^4^ Cut-off defined considering the WHO recommendation of at least 75 min/week of vigorous physical activity [20]. ^5^ Number of observations is lower than 776 due to missing data.

**Table 3:** Prevalence and corresponding 95% confidence interval of being overweight/obese, abdominal obesity, and hypertension in a sample of young adults aged 18–25 years from Mozambique; ORs and 95% confidence intervals of the association between sociodemographic characteristics and the previous variables are included.

	Overweight/Obesity ^2,3^		Abdominal Obesity ^3,4^		Hypertension ^5^	
	% (95%CI)	OR ^1^ (95%CI)	% (95%CI)	OR ^1^ (95%CI)	% (95%CI)	OR ^1^ (95%CI)
Sex ^6^						
Women	17.6 (13.0–23.2)	ref	2.5 (1.3–4.6)	ref	14.1 (10.8–18.4)	ref
Men	8.6 (4.9–15.0)	0.43 (0.21–0.87)	---	---	21.0 (14.8–28.9)	2.09 (1.20–3.63)
Place of residence ^6^						
Rural	10.7 (7.3–15.4)	ref	1.1 (0.3–3.5)	ref	18.0 (13.2–24.1)	ref
Urban	18.0 (12.8–24.5)	1.57 (0.73- 3.39)	1.9 (1.0–3.7)	1.20 (0.35–4.10)	15.0 (11.2–20.0)	0.97 (0.49–1.93)
Education (years) ^6^						
None	9.8 (4.8–19.2)	ref	2.3 (0.5–8.5)	ref	24.4 (16.1–35.0)	ref
1–7	11.7 (7.1–18.5)	1.07 (0.40–2.86)	0.7 (0.2–2.2)	0.29 (0.09–0.93)	18.1 (13.4–24.1)	0.41 (0.19–0.89)
>8	17.6 (13.0–23.5)	1.26 (0.41–3.87)	2.0 (1.1–3.9)	0.76 (0.21–2.79)	12.5 (8.4–18.0)	0.24 (0.11–0.54)
Monthly income (USD/household member aged ≥18 years) ^6^						
0–15	10.8 (6.6–17.4)	ref	0.8 (0.1–5.8)	ref	14.3 (9.4–21.0)	ref
16–44	15.0 (10.3–21.2)	1.29 (0.67–2.48)	2.3 (1.2–4.6)	2.58 (0.44–15.26)	21.0 (14.9–28.5)	1.75 (0.97–3.18)
≥45	20.2 (14.0–28.3)	1.84 (0.95–3.57)	2.1 (0.8–5.1)	2.47 (0.37–16.71)	17.3 (11.9–24.6)	1.65 (0.92–2.97)

ref: Reference category. ^1^ Derived from models including sex, place of residence, education, and income. ^2^ Defined as BMI ≥ 25.0 kg/m^2^ [21]. ^3^ Defined as a waist circumference greater than 88 cm and 102 cm in women and men, respectively [21]. ^4^ Pregnant women were excluded from this analysis. ^5^ Defined as SBP of at least 140 mmHg, DSP of at least 90 mmHg, or antihypertensive drug therapy in the last two weeks, or any combination of these indicators [22]. ^6^ The number of observations is lower than 776 due to missing data.

## Data Availability

The data presented in this study are available on request to the corresponding author. The data are not publicly available due to restrictions in the informed consent to specific scientific objectives.
